# Placental immunopathology and congenital transmission of Zika virus and *Trypanosoma cruzi*: comparative mechanisms, immune disturbances, and potential co-infection scenarios

**DOI:** 10.3389/fimmu.2026.1824065

**Published:** 2026-10-07

**Authors:** Carlos Andres Rodriguez-Salazar, Delia Piedad Recalde-Reyes, Nisha J. Garg, Maria I. Giraldo

**Affiliations:** 1 Molecular Biology and Virology Laboratory, Faculty of Medicine and Health Sciences, Corporación Universitaria Empresarial Alexander von Humboldt, Armenia, Colombia; 2 Department of Microbiology & Immunology, University of Texas Medical Branch (UTMB), Galveston, TX, United States; 3 Institute for Human Infections and Immunity (IHII), UTMB, Galveston, TX, United States

**Keywords:** coinfection, congenital infection, fetal immune development, immunopathology, maternal–fetal interface, placental Immunity, *Trypanosoma cruzi*, Zika virus

## Abstract

Congenital transmission of Zika virus (ZIKV) and *Trypanosoma cruzi* (*T. cruzi*) remains a major public health concern in the Americas. Both pathogens cross the placental barrier and cause severe neonatal outcomes, yet no confirmed congenital co-infections have been reported, and their combined effects on maternal–fetal health are unknown. Critical gaps persist regarding placental immunopathology, fetal immune programming, and long-term consequences of prenatal exposure. This review synthesizes current evidence on the routes and mechanisms of placental transmission of ZIKV and *T. cruzi*, their tropism for placental and fetal tissues, and their distinct immunological footprints. We summarize how congenital infection perturbs fetal immune maturation, including dysregulation of type I/type III interferons, Th1-skewed inflammation, altered immune-tolerance pathways involving HLA-G and PD-1/PD-L1 signaling, and early innate imprinting. We review advances in vaccine development (spanning inactivated, DNA, mRNA, subunit, and viral vector platforms) and highlight barriers such as antibody-dependent enhancement in ZIKV vaccines and the scarcity of pregnancy-relevant preclinical models for *T. cruzi*. Promising candidates with evidence of fetal or maternal protection in animal studies are described. Therapeutic approaches, including antiviral discovery, immunomodulatory strategies, and emerging anti-*T. cruzi* compounds are also examined. A detailed understanding of the immunopathology of congenital ZIKV and *T. cruzi* infections is essential to improve diagnostic accuracy, preventive strategies, and maternal–fetal clinical care. Integrated mechanistic and translational research is urgently needed to assess the plausibility of co-infection, identify biomarkers of fetal risk, and guide the development of pregnancy-safe vaccines and therapeutics, particularly in resource-limited, endemic settings.

## 1 Introduction

Infections during pregnancy have significant and potentially devastating consequences for both the pregnant mother and the developing fetus. Congenital infections are a major contributor to fetal and neonatal morbidity and mortality. A wide range of maternal infections can lead to severe fetal outcomes, including congenital anomalies in various organ systems, growth restriction, stillbirth, miscarriage, neonatal death, and disrupted organ development. It can also result in health complications for the mother. Pathogens of the TORCH group (*Toxoplasma gondii*, Rubella virus, Cytomegalovirus, and Herpes Simplex Virus) are known to be transmissible congenitally. *Zika virus* (ZIKV) and *Trypanosoma cruzi* (*T. cruzi*) have recently been recognized to impose a significant risk of congenital transmission in the Americas (1, 2).

ZIKV is a positive-sense RNA virus of the *Flavivirus* genus, initially identified in Africa and first detected in the Pacific in 2007 (Yap Island, Micronesia), followed by major epidemics in French Polynesia (2013–2014) and subsequent emergence in the Americas during 2015–2016 (3). Transmitted mainly by *Aedes* mosquitoes, ZIKV can also spread through sexual contact, blood exposure, and vertical transmission. Mosquito-borne transmission has been reported in 89 countries, including the United States, where autochthonous ZIKV transmission has been documented in Florida and Texas, highlighting the capacity of ZIKV to establish local transmission under suitable environmental conditions (4–7). ZIKV infection during pregnancy can cause congenital Zika syndrome (CZS), characterized by microcephaly and other severe neurological, ocular, and musculoskeletal abnormalities. Cohort studies have reported ZIKV-associated birth defects in approximately 6–7% of exposed pregnancies overall, with estimates increasing to approximately 11–13% following first-trimester infection (3, 8, 9). No approved antiviral treatments are currently available, and although several vaccine candidates have entered early-phase clinical trials, no licensed ZIKV vaccine exists to date (10).

In contrast, *T. cruzi* is a unicellular protozoan that causes Chagas disease (CD). Transmission primarily occurs via triatomine insects (kissing bugs) in endemic areas; alternative routes include blood transfusion, organ transplantation, laboratory accidents, and oral ingestion. CD remains a major public health concern in Latin America, affecting an estimated ~6 million people with an additional ~71 million at risk of infection (11, 12). Recent epidemiological studies demonstrate that CD is not only imported but also prevalent in parts of the United States, supported by locally acquired cases and the presence of the competent vectors (13). The USA alone accounts for over 300,000 cases, and recent analyses estimate approximately 10,000 locally acquired infections, primarily concentrated in southern states such as Texas, Louisiana, and Arizona (14). Additionally, reports from the Washington, D.C. metropolitan area have revealed seropositive individuals with no travel history to Latin America (15). Besides, Spain now represents the European country with the highest burden of CD, largely driven by migration from endemic regions (16). Collectively, these findings support the view that Chagas disease should be regarded as a neglected yet emerging endemic infection in the United States and Europe, shaped primarily by human migration and socioeconomic factors, together with the presence of competent vectors in some regions.

*T. cruzi* can also be transmitted from mother to child, sustaining the infection cycle in the absence of vectors (17). In this context, vertical transmission of *T. cruzi* refers primarily to transplacental infection (congenital Chagas disease), although intrapartum exposure may also occur through maternal blood at delivery. Approximately 2 million women of reproductive age are infected globally, with congenital transmission occurring in 5–10% of pregnancies, with risk influenced by maternal parasitemia and immune status and potentially increased in acute or reactivated infections (18, 19). Benznidazole is recommended before conception, as no antiparasitic therapies are currently approved for use during pregnancy. Congenitally infected neonates may present with low birth weight, hepatosplenomegaly, respiratory distress, myocarditis, or meningoencephalitis (20, 21). Some studies indicate up to 29% of congenitally infected infants develop acute cardiac involvement, and survivors remain at long-term risk of chronic cardiomyopathy and digestive or neurological complications later in life (21–23).

Although ZIKV and *T. cruzi* employ fundamentally different biological strategies (viral versus parasitic), their capacity to breach maternal–fetal barriers places the placenta at the center of pathogenesis. Both pathogens exhibit tropism for placental tissues, induce local inflammation, and modulate immune tolerance mechanisms, yet the immunopathological pathways underlying their transplacental passage remain incompletely understood. Furthermore, although ZIKV and *T. cruzi* overlap geographically in parts of Latin America, to our knowledge, no well-documented case(s) of congenital co-infection have been reported. Geographic overlap creates an epidemiological opportunity for maternal co-exposure but does not establish that congenital co-infection occurs or that the two pathogens interact biologically at the maternal–fetal interface. Whether the absence of reported cases reflects true rarity, differences in exposure patterns, limited surveillance, diagnostic constraints, or other factors remains unknown and represents an important gap in maternal–fetal infectious disease research.

Understanding how ZIKV and *T. cruzi* independently affect placental structures and maternal–fetal immunity is essential for improving prevention, diagnosis, and fetal outcomes. Accordingly, this review summarizes current evidence on the placental immunopathology and congenital transmission mechanisms of ZIKV and *T. cruzi*, compares their distinct pathways of maternal–fetal invasion and immune disturbances at the maternal–fetal interface, reviews advances in vaccine and therapeutic development, and examines potential, biologically plausible co-infection scenarios to identify key knowledge gaps and future research priorities.

## 2 Search strategy and selection criteria

Because direct evidence addressing congenital ZIKV–*T. cruzi* co-infection is not documented in the literature, this study was designed as a narrative review to integrate available evidence on the biology, placental pathogenesis, immunology, clinical manifestations, and preventive and therapeutic approaches associated with each pathogen. The search therefore aimed to provide a broad mechanistic and translational synthesis rather than to perform a systematic review or quantitative evidence synthesis.

For terminological consistency throughout this review, “congenital transmission” refers specifically to pathogen transmission to the fetus during gestation, including transplacental infection, whereas “vertical transmission” is used as a broader term for mother-to-child transmission and may also encompass intrapartum and, where applicable, postnatal routes. “*In utero* infection” is used descriptively for fetal infection acquired during gestation.

Literature searches were conducted in PubMed and ScienceDirect, initially covering publications from 2010 to 2025 to capture key mechanistic and epidemiological studies generated during and after the major ZIKV outbreaks, as well as recent advances in congenital Chagas disease research. During manuscript revision, targeted searches were updated through September 2026 to incorporate newly published evidence and address specific issues raised during peer review. We also consulted ClinicalTrials.gov to verify the current development status of relevant vaccine candidates and selected therapeutics that had entered clinical evaluation. We also included earlier seminal publications when needed to contextualize placental immunopathology, congenital transmission, fetal and neonatal immune responses, or the historical development of vaccine and therapeutic approaches.

Searches used combinations of free-text terms and, when applicable, MeSH headings, including “Zika virus,” “*Trypanosoma cruzi*,” “congenital infection,” “vertical transmission,” “maternal–fetal interface,” “placenta,” “coinfection,” “immunopathology,” “fetal immune development,” “vaccine development,” and “therapeutic strategies.” Eligible publications included original research articles, reviews, epidemiological studies, clinical reports, and relevant preclinical studies published in English or Spanish that addressed one or more of the major themes listed above. We also manually screened reference lists of relevant publications to identify additional studies and seminal reports not captured by the primary searches.

Studies were excluded when they were unrelated to the major themes of the review, did not provide relevant mechanistic, clinical, epidemiological, or translational information, or were not available as full-text publications. Because the literature search was conducted iteratively for a narrative synthesis rather than according to a prospectively defined systematic-review protocol, we did not maintain formal records of the total number of articles retrieved, screened, and excluded. Consequently, no PRISMA-style study-flow analysis or formal risk-of-bias assessment was performed. We acknowledge that this approach may introduce selection bias, and this limitation should be considered when interpreting the synthesis presented here. To mitigate this limitation, we sought to incorporate evidence from different study designs and both pathogens and to distinguish established pathogen-specific findings from proposed mechanisms and hypothesis-generating co-infection scenarios.

Throughout the review, we distinguish among (i) established mechanisms supported by direct experimental or clinical evidence for either pathogen, (ii) proposed mechanisms supported by indirect or incomplete pathogen-specific evidence, and (iii) hypothetical ZIKV–*T. cruzi* interactions extrapolated from independently characterized mechanisms. The latter are explicitly presented as hypothesis-generating and not as demonstrated consequences of co-infection.

## 3 Routes and mechanisms of congenital transmission

### 3.1 ZIKV: placental tropism and transmission

ZIKV infection in pregnancy unfolds as a stepwise process that begins in the maternal bloodstream and progressively advances towards the fetal compartment. Shortly after maternal viremia develops, the virus reaches the placenta hematogenously and encounters the first layers of the maternal–fetal interface. Clinical reports detecting ZIKV RNA and antigens in amniotic fluid, chorionic villi, placental biopsies, and fetal neural tissue confirm that the virus can cross the placental barrier, which is consistent with its role in congenital malformations (24). At the placental interface, the virus interacts with multiple cell types. Experimental studies using primary trophoblasts and human placental explants show that ZIKV readily infects cytotrophoblasts (CTBs) and extravillous trophoblasts (EVTs) - the cells responsible for anchoring the placenta and invading the maternal decidua (25–27). These placental cell populations express ZIKV attachment and entry cofactors, including the TAM-family receptor tyrosine kinases AXL and TYRO3, the phosphatidylserine-binding receptor TIM-1, and the C-type lectin DC-SIGN. Studies using primary human placental cells have shown that ZIKV can infect trophoblasts, endothelial cells, fibroblasts, and Hofbauer cells that express one or more of these factors, supporting a potential facilitating role in viral attachment or entry. However, receptor expression does not establish an absolute requirement for infection. Genetic studies in mice have demonstrated that ZIKV infection of the placenta and fetus, as well as mother-to-fetus transmission, can occur in the absence of AXL or other TAM receptors, indicating substantial redundancy in ZIKV entry pathways (28). Accordingly, AXL and TYRO3 should be considered facilitating factors rather than indispensable receptors for placental infection or congenital transmission (29, 30).

Although the syncytiotrophoblast (STB) normally constitutes a major barrier to invading pathogens, placental susceptibility to ZIKV varies with gestational age, trophoblast differentiation state, local interferon signaling, and viral lineage, with first-trimester placental tissues remaining susceptible to infection despite intrinsic antiviral defenses (31, 32). Once inside trophoblast layers, ZIKV triggers the production of type I interferons and inflammatory cytokines, disrupting tight junctions and weakening the integrity of the villous surface (29, 33–36). This inflammatory microenvironment opens paracellular routes and compromises the cohesive architecture that normally protects the fetus. The virus then gains access to the villous core, where Hofbauer cells (resident fetal macrophages) are highly susceptible. Infected Hofbauer cells serve as reservoirs and may facilitate viral migration within the stroma (27). As the infection progresses, endothelial cells lining fetal capillaries within the villi can become infected or injured. ZIKV-induced apoptosis, capillary rarefaction, and endothelial disruption increase vascular permeability and allow virions to reach the fetal bloodstream. Once in fetal circulation, ZIKV displays marked neurotropism: it preferentially targets neural progenitor cells in the developing brain, impairing neurogenesis, cortical layering, and axonal organization (25, 26). These combined events (progressive breach of trophoblast barriers, dissemination through infected macrophages, and eventual neuroinvasion) form a stepwise mechanistic pathway leading to congenital Zika syndrome (**Figure 1**).

**Figure 1:**
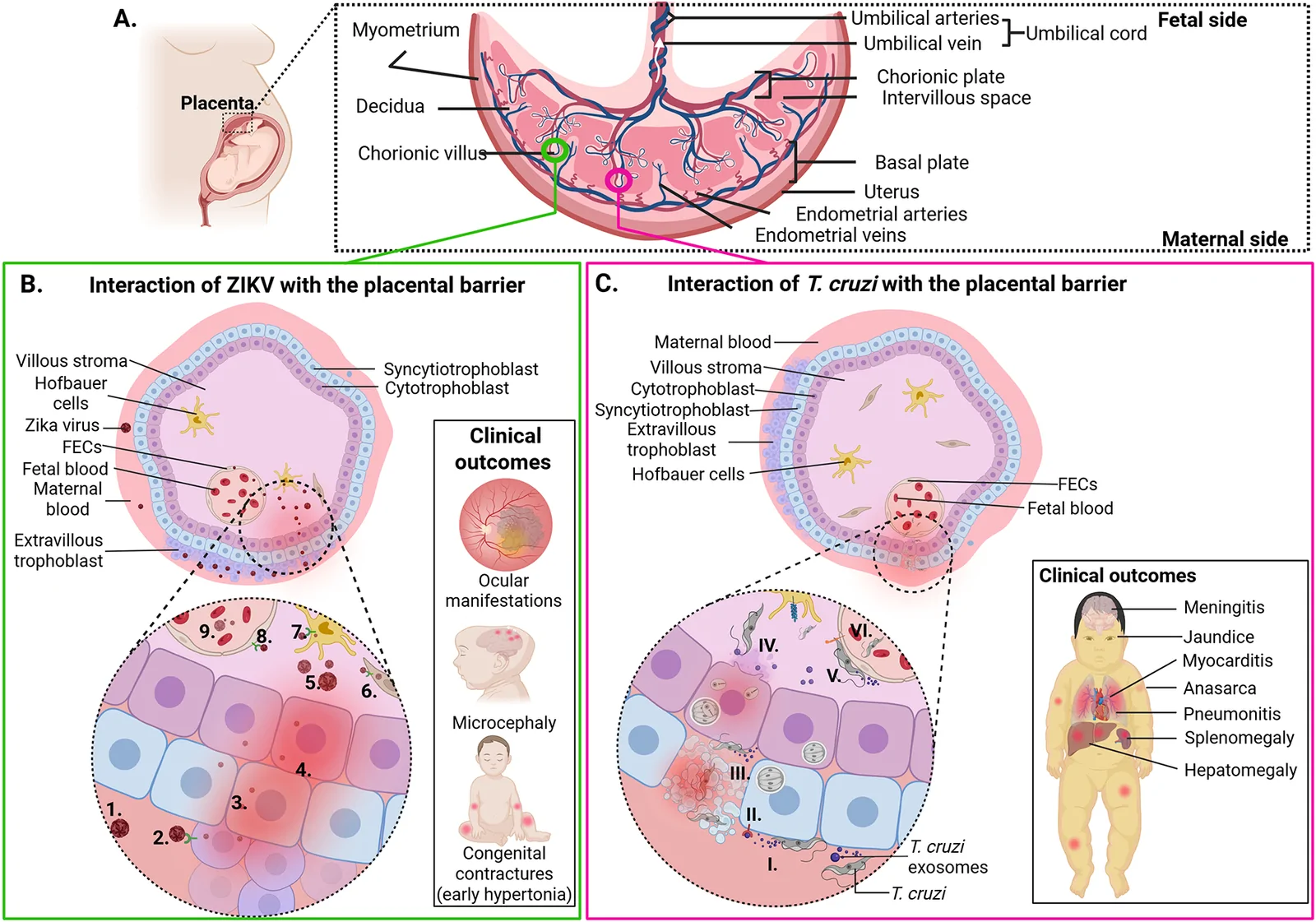
Mechanisms of congenital infection and clinical manifestations caused by ZIKV and (T) cruzi. **(A)** Anatomy of the placenta. General structure showing maternal and fetal sides. Maternal blood flows into the intervillous space, while fetal blood circulates in the chorionic villi through fetal vessels. **(B)** Cross-section of the placental barrier to show ZIKV infection mechanism and clinical manifestations. 1. ZIKV in the maternal blood within the intervillous space. 2. ZIKV encounters extravillous trophoblasts (EVTs), which express several cofactors, including AXL, TYRO3, TIM-1, and lectin receptors that can promote viral attachment and entry. 3. ZIKV reaches trophoblast layers, with susceptibility varying according to trophoblast subtype, gestational age, and local antiviral signaling. 4. Viral replication and infection-associated inflammation can compromise trophoblast integrity and placental barrier function. 5. ZIKV can infect cytotrophoblasts (CTBs) and other susceptible stromal populations, allowing progression into deeper villous compartments. 6. Hofbauer cells are permissive to ZIKV infection and may contribute to viral persistence and dissemination within the villous stroma. 7. Fetal endothelial cells can also support ZIKV infection, providing a potential route toward the fetal vascular compartment. 8. Disruption of endothelial and placental barrier integrity may facilitate viral access to the fetal circulation. 9. Viral spread to the fetal brain, infecting neural progenitor cells and impairing neurogenesis, leading to cortical thinning and subcortical calcifications. 10. Clinical features of Congenital Zika Syndrome (CZS): severe microcephaly, ocular lesions (e.g., macular scarring), and congenital contractures with hypertonia. **(C)** Cross-section of the placental barrier illustrating *T. cruzi* infection mechanism and clinical manifestations (I-VII). (I) Circulating trypomastigotes interact with laminin and fibronectin on the STB surface. II. Parasite-derived exosomes activate TLR2/TLR9 on the STB, inducing inflammation and weakening barrier integrity. III. Focal damage to STB permits parasite permeation into deeper villous layers. IV. Trypomastigotes cross the CTB and infect Hofbauer cells, where intracellular replication occurs. (V) Parasites invade or traverse fetal endothelial cells, disrupting endothelial integrity and facilitating access to the fetal circulation. VI. Dissemination into fetal tissues results in systemic infection. VII. Clinical manifestations of congenital Chagas disease include myocarditis, hepatomegaly, splenomegaly, pneumonitis, meningoencephalitis, jaundice, low birth weight, and anasarca. Created in BioRender. Rodriguez salazar, C. A. (2026) https://BioRender.com/0jl99gs.

### 3.2 *T. cruzi*: placental invasion and maternal–fetal passage

Congenital transmission of *T. cruzi* can occur during any trimester of pregnancy but is more likely after the placental intervillous space is established (~10–12 weeks of gestation), and risk increases during 22–26 weeks of gestation (1, 37). Transmission rates average 5–10% of pregnancies in chronically infected women and rise sharply in acute or reactivated infections, particularly under immunosuppressive conditions (19, 38–43). As mentioned above, the maternal–fetal interface consists of decidua, villous trophoblasts (CTB and STB), extravillous trophoblasts (EVT), Hofbauer cells, and fetal endothelial cells. Some studies show that *T. cruzi* trypomastigotes adhere to and invade STB and CTB by interacting with extracellular matrix components such as laminin and fibronectin, as well as galectins and heparan sulfate proteoglycans (20, 44). Histological analyses of placentas from congenitally infected newborns or stillbirths have shown *T. cruzi* amastigote nests, parasite infiltration of the trophoblastic barrier, and parasitism of stromal and Hofbauer cells (44). Lesions were most prominent near the marginal sinus and decreased towards the chorionic plate, likely reflecting regional differences in trophoblast thickness and vascular architecture, which may favor parasite infiltration and predispose to membrane rupture or preterm delivery (45, 46). *Ex vivo* placental explant studies demonstrated that parasite invasion induces villous disorganization, trophoblast detachment, and syncytial layer disruption accompanied by local inflammation (47–50). Parasite-derived extracellular vesicles activated toll-like receptors (TLR2 and TLR9), amplifying inflammation and weakening the syncytial barrier, facilitating parasite traversal (50). Once within the villous stroma, trypomastigotes infect Hofbauer and fetal endothelial cells, allowing hematogenous dissemination into the fetal circulation (44, 47, 51).

Alternative transmission routes may include fetal ingestion of amniotic fluid containing parasites or exposure to maternal blood during delivery when placental tears occur (19, 39, 51). These processes support a model in which congenital *T. cruzi* transmission results from a combination of maternal parasitemia, placental inflammation, and direct cellular invasion mechanisms that compromise the structural and immunological integrity of the placental barrier (**Figure 1**).

### 3.3 Comparative mechanisms and conceptual nodes of overlap

Although ZIKV and *T. cruzi* use fundamentally different biological strategies (viral replication versus intracellular parasitism), their processes of congenital transmission share convergent features at the maternal–fetal interface. Both pathogens must survive in the presence of the maternal immune system, breach trophoblast barriers, and infiltrate villous stromal and endothelial compartments before reaching the fetal circulation (17, 25, 52). These parallel requirements create mechanistic intersections that may illuminate how each pathogen exploits placental vulnerabilities.

First, trophoblast susceptibility and barrier disruption constitute a shared gateway. ZIKV readily infects CTB and EVT layers (25, 27, 35, 53), whereas *T. cruzi* adheres to and invades STB and CTB cells through interactions with extracellular matrix components and surface glycoconjugates (17, 47, 50, 54). Despite differences in cell entry mechanisms, both pathogens induce tight-junction disorganization, syncytial thinning, and focal disruption of trophoblast layers, facilitating paracellular or transcellular passage (35, 47, 49, 54). Inflammasome and interferon-driven damage during ZIKV infection (34, 36, 55) parallels TLR2/TLR9-mediated inflammation and syncytial weakening during *T. cruzi* infection (50, 56), highlighting a shared inflammatory mechanism that reduces placental barrier resilience.

Second, both pathogens target Hofbauer cells, a critical point of overlap. ZIKV-infected Hofbauer cells serve as reservoirs and may facilitate viral migration toward fetal vessels (27, 35, 53), whereas *T. cruzi* amastigotes replicate within Hofbauer cells and stromal macrophages, contributing to villous injury and subsequent dissemination of the parasite in fetal organs (17, 47, 57). Third, endothelial involvement is a convergent hallmark. ZIKV induces apoptosis and thinning of fetal capillaries (35, 53, 58), while *T. cruzi* disrupts endothelial structures through direct invasion and inflammatory injury (47, 49, 59). In both infections, endothelial permeability increases, enabling hematogenous spread into fetal circulation.

Despite these mechanistic intersections, important divergences remain. ZIKV exhibits marked neurotropism, with congenital disease driven largely by infection of neural progenitors following placental breach (26, 60). In contrast, *T. cruzi* pathogenesis results from intracellular replication and sustained inflammation, primarily affecting the fetal heart (17, 22). Transmission determinants also differ: ZIKV depends heavily on gestational timing and local interferon tone (25, 34, 61), whereas *T. cruzi* congenital transmission correlates strongly with maternal parasitemia and immune suppression (17, 37, 62).

Importantly, the similarities described above derive from independent studies of ZIKV or *T. cruzi* infection and should not be interpreted as evidence of direct interaction between the two pathogens. To date, no confirmed cases of congenital ZIKV–*T. cruzi* co-infection have been reported, and no experimental studies have directly examined simultaneous infection at the maternal–fetal interface. Accordingly, the shared involvement of trophoblast barriers, Hofbauer cells, inflammatory pathways, and fetal endothelial compartments identifies potential points of mechanistic convergence rather than established mechanisms of co-infection. These observations provide a conceptual, hypothesis-generating framework for the potential interactions discussed in Sections 4.3 and 5.3, which will require direct experimental and clinical validation.

## 4 Clinical manifestations of congenital infection

### 4.1 ZIKV: fetal and neonatal outcomes

ZIKV infection during pregnancy can lead to a spectrum of congenital abnormalities collectively referred to as CZS. The severity of fetal injury depends strongly on the timing of maternal infection, with first- and second-trimester exposure associated with most severe outcomes (8, 58). Clinical and imaging studies of affected fetuses and newborns have identified hallmark neurological and systemic features of ZIKV infection. The most characteristic finding is severe microcephaly, often accompanied by cortical thinning, ventriculomegaly, subcortical calcification, and cerebral atrophy due to ZIKV-induced destruction of neural progenitor cells and disruption of neuronal differentiation (8). Gross cranial deformities include a partially collapsed skull, redundant scalp skin, and prominent occipital bone. Additional central nervous system (CNS) abnormalities may include lissencephaly, cerebellar hypoplasia, and corpus callosum dysgenesis (8, 60).

Ocular involvement is one of the most frequent manifestations of CZS, reported in up to 55% of the infants exposed to ZIKV infection (63). The ocular pathology follows a pattern that mirrors fetal neurotropism: once ZIKV reaches the developing eye, it targets retinal progenitor cells and neuroretina, triggering inflammatory and degenerative changes that progressively shape the spectrum of lesions observed postnatally. Typical findings include macular scarring, pigmentary retinopathy, optic nerve hypoplasia or pallor, and varying degrees of chorioretinal atrophy, which can disrupt visual pathways and result in permanent visual impairment (63).

Musculoskeletal abnormalities, particularly arthrogryposis, arise through a similar mechanism of neurogenic injury. Rather than originating from primary defects in bone or muscle, these contractures are thought to reflect chronic denervation resulting from ZIKV-induced damage of anterior horn cells, peripheral motor neurons, and corticospinal tract pathways. This disruption of fetal motor circuitry impairs normal muscle innervation, leading to fixed limb postures and joint contractures that are frequently observed in severe CZS cases (8, 58, 60).

Although less frequent than CNS abnormalities, several extra-neurological manifestations have been documented in infants with CZS, reflecting the systemic impact of gestational ZIKV infection (58). Transient myocardial dysfunction or mild cardiomegaly has been observed in a small subset of ZIKV-infected neonates, typically presenting as reversible left ventricular dilation or mild systolic dysfunction without structural defects (64, 65). Cardiac function usually normalizes during follow-up, indicating a self-limited process rather than chronic cardiomyopathy. Pulmonary hypoplasia, though uncommon, has also been described in severe congenital cases of ZIKV infection (66). This condition is believed to result from prolonged oligohydramnios and impaired fetal thoracic expansion due to neurological compromise, such as brainstem dysfunction or decreased fetal movement. Consequently, affected infants may develop respiratory failure immediately after birth despite the absence of intrinsic pulmonary infection. Hepatic involvement in CZS is rarely detected; a few infants demonstrate mild and transient elevations in transaminases, yet clinically significant hepatomegaly or cholestasis is unusual. Hepatic alterations are thought to arise from systemic inflammatory or ischemic injury related to placental insufficiency, rather than from productive viral replication, positioning the liver as a secondary target in the setting of compromised fetal–placental circulation (58).

Hydrops fetalis represents one of the most severe and exceptional manifestations of congenital infection (67). In these cases, profound placental insufficiency combined with fetal cardiac dysfunction can lead to generalized edema, ascites, and pleural or pericardial effusions. These presentations represent rare, severe outcomes within the spectrum of congenital ZIKV infection.

In summary, although CZS is clinically defined by its neurological manifestations, severe *in utero* ZIKV infection can disturb several fetal organ systems through a combination of placental insufficiency, circulatory instability, and neurogenic dysfunction. These mechanisms broaden the clinical spectrum beyond the brain and highlight the need for systematic neonatal assessments beyond the neuroimaging to include cardiac, respiratory, hepatic, and ophthalmologic evaluations (64). Even so, infants who appear asymptomatic at birth may still harbor subtle neurological or retinal alterations, reinforcing the clinical value of structured postnatal follow-up for all newborns with confirmed *in utero* ZIKV exposure.

### 4.2 *T. cruzi*: fetal and neonatal outcomes

Congenital *T. cruzi* infection represents one of the principal mechanisms sustaining Chagas disease transmission in the absence of vectors, particularly in areas where vectorial control programs have been successful. The probability of fetal infection increases if maternal parasitemia is high, which occurs in acute or reactivated infections. It is documented that risk of fetal infection can reach up to 53% when women acquire infection during pregnancy or experience immunosuppression (17, 19, 38–40). Chronic infection with low rate of parasite replication during pregnancy is, in general, associated with lower transmission rates, estimated at 5–10% (18, 19). Congenital infection can occur in any trimester, but the risk becomes more significant after the establishment of the placental intervillous space (around 12 weeks of gestation), which facilitates parasite access to fetal tissues (37).

Clinical manifestations in congenitally infected neonates are highly variable, ranging from asymptomatic infection to severe multisystemic disease. Approximately 60–70% of infected newborns are asymptomatic at birth but may develop detectable parasitemia, anemia, or hepatosplenomegaly within the first few weeks of life (37). Symptomatic neonates frequently present with low birth weight, prematurity, hepatomegaly, splenomegaly, jaundice, and anemia, reflecting systemic inflammation and hepatic dysfunction secondary to parasite replication in the reticuloendothelial system (20, 21). Cardiac involvement is one of the most characteristic findings, with reported rates between 10% and 30% among symptomatic cases (21). Affected infants may develop myocarditis, arrhythmias, or signs of heart failure, while echocardiography can reveal ventricular dilation or reduced contractility. Most cases respond to antiparasitic therapy, but untreated infants risk progression to chronic cardiomyopathy later in life (21–23).

Neurological manifestations occur in approximately 5–10% of congenital cases of *T. cruzi* infection, and are usually associated with meningoencephalitis, presenting as irritability, seizures, or hypotonia. Severe CNS disease may coincide with high parasitemia and extensive inflammatory infiltration of the meninges and choroid plexus, although this is rarely detected during clinical evaluation of newborns. Respiratory distress and pneumonitis have also been reported, probably secondary to inflammatory edema or myocardial compromise (21). Gastrointestinal symptoms, such as feeding intolerance or abdominal distension, are uncommon in congenital cases and typically mild when present (52, 62).

Unlike congenital viral infections, ocular involvement is uncommon in congenital CD, though isolated reports describe chorioretinitis or uveitis in neonates with high parasite loads (68). Hydrops fetalis and anasarca have occasionally been reported in severely infected fetuses, likely related to cardiac failure or hepatic dysfunction rather than direct placental damage (69, 70). Importantly, vertical transmission is not always accompanied by overt placental pathology; many infected placentas show only mild villitis or focal necrosis, suggesting that parasite passage may occur without major tissue destruction (71).

Overall, the prognosis of congenital *T. cruzi* infection depends on early detection and prompt treatment. When therapy with benznidazole or nifurtimox is initiated during the neonatal period, the cure rate exceeds 90%, and long-term sequelae are rare (20, 71, 72). In contrast, untreated infants risk developing chronic infection with potential cardiac, digestive, or neurological complications in adulthood. Therefore, universal screening of pregnant women and newborns in endemic and migration-affected regions is essential to interrupt congenital transmission and reduce Chagas disease burden (52, 62).

### 4.3 Hypothetical clinical scenarios and knowledge gaps in co-infection

Despite geographic overlap between areas affected by ZIKV and *T. cruzi* in Latin America, no confirmed cases of congenital co-infection have been reported to date. Importantly, geographic co-endemicity indicates an epidemiological opportunity for maternal co-exposure but does not, by itself, provide evidence of congenital co-infection or mechanistic interaction between the two pathogens. The reasons for the absence of documented cases remain unknown and may include true rarity of simultaneous infection during pregnancy, differences in the spatial and temporal patterns of pathogen circulation, limited surveillance, diagnostic constraints, or under-recognition of dual infection. Both pathogens circulate in settings characterized by vector exposure, socioeconomic vulnerability, and limited access to prenatal care, conditions under which congenital infections may remain undetected.

Diagnostic limitations may contribute to under-recognition of dual infection, although the extent of this contribution is unknown. For ZIKV, nucleic acid amplification testing provides highly specific evidence of current or recent infection, but the diagnostic window for viral RNA in serum is often limited, and a negative molecular result does not exclude recent infection; prolonged RNA detection has nevertheless been reported in some pregnant individuals. Serological diagnosis is further complicated by extensive antibody cross-reactivity with other flaviviruses, particularly dengue virus, and even neutralization assays may not reliably discriminate among flavivirus infections in individuals with prior flavivirus exposure (73, 74). In maternal Chagas disease, parasitemia is generally low and intermittent, which limits the sensitivity of direct parasitological and single-time-point molecular detection. Serial blood sampling can substantially increase PCR sensitivity, illustrating the temporal variability of circulating parasite DNA. Diagnosis of congenital *T. cruzi* infection likewise depends on specimen type and sampling time, and early molecular or parasitological testing may require repeated evaluation before infection can be excluded (23, 75, 76).

These pathogen-specific limitations are particularly relevant when considering potential co-infection. Although multiplex molecular platforms have been developed for arboviral diagnosis and broader febrile-illness surveillance, to our knowledge, no clinically validated diagnostic algorithm or dual-pathogen molecular assay has been established specifically for the simultaneous detection of ZIKV and *T. cruzi* in placental tissue or cord blood. Access to molecular diagnostics is also uneven in many endemic and resource-limited settings. Consequently, diagnostic constraints make under-recognition of congenital co-infection plausible, but do not establish that such co-infection occurs at an appreciable frequency.

Evidence from other *T. cruzi* co-infection settings provides useful context for hypothesis generation for congenital ZIKV–*T. cruzi* co-infection. In people living with HIV, profound cellular immunosuppression is strongly associated with reactivation of chronic *T. cruzi* infection, demonstrating that substantial changes in host immune status can modify parasite control. Co-infections involving *T. cruzi* and arboviruses, including dengue and chikungunya, have also been documented in co-endemic populations; though these studies did not examine whether arbovirus infection modifies the severity or pathogenesis of Chagas disease. Collectively, these observations demonstrate that dual infection can occur and that the immunological environment generated by a second infection may influence *T. cruzi* control, but they do not predict the direction or magnitude of a potential interaction with ZIKV during pregnancy (77, 78).

From a biological perspective, ZIKV and *T. cruzi* employ distinct but partially convergent mechanisms to invade the placenta and modulate the local immune environment. ZIKV attachment and entry can occur via multiple cofactors in susceptible placental cells, ZIKV independently induces trophoblast stress, apoptosis, cytoskeletal alterations, and endothelial injury during placental infection (28, 79). In contrast, *T. cruzi* traverses the maternal–fetal interface through extracellular matrix degradation, inflammatory activation, and infection of Hofbauer cells (12, 17, 27, 30, 33, 34, 44–46, 55, 80, 81). Although no studies have evaluated simultaneous exposure of placental tissues to both pathogens, and available animal models do not adequately reproduce human placentation (82, 83), these independently characterized mechanisms raise testable questions regarding whether simultaneous infection could modify placental susceptibility or fetal injury. For example, ZIKV-induced disruption of the STB could potentially increase placental accessibility to *T. cruzi*, whereas parasite-driven inflammation might alter local antiviral responses and thereby influence ZIKV replication or dissemination. These possibilities should be regarded as mechanistic hypotheses for future investigation.

Potential effects on neonatal immune development are also biologically plausible given the pathogen-specific immune signatures described above. Independently, both infections have been associated with elevated levels of proinflammatory cytokines such as IL-6, TNF-α, and IFN-γ in cord blood and with evidence of early innate activation (8, 50, 61, 84–86). Thus, simultaneous exposure to antiviral and antiparasitic inflammatory signals could influence fetal immune programming or placental immune homeostasis. The combination of ZIKV-associated modulation of antiviral and trophoblast responses with Th1-skewed inflammatory response caused by *T. cruzi* infection provides a rationale for integrated maternal–fetal studies using paired pathogen detection and immunological profiling across placental, maternal, and neonatal compartments (8, 51, 58, 80, 81, 84, 87).

From a clinical perspective, congenital co-infection could conceivably produce additive, synergistic, antagonistic, or independent disease phenotypes. A composite presentation might include manifestations associated with CZS, such as microcephaly, ventriculomegaly, or ocular abnormalities, together with systemic manifestations reported in congenital Chagas disease, including hepatosplenomegaly, myocarditis, or anemia. Though, such a phenotype has not been documented, this scenario illustrates the diagnostic complexity that could arise if both infections occurred in the same pregnancy. A comparative summary of the principal neonatal manifestations associated with congenital ZIKV and T. cruzi infections is provided in **Table 1**.

**Table 1:** Neonatal manifestations associated with congenital infections by the Zika virus and *T. cruzi*.

Neonatal symptoms	Common to ZIKV and *T. cruzi*	Specific to ZIKV	Specific to *T. cruzi*	Key references ZIKV *T. cruzi*	
Low birth weight	Due to placental insufficiency or systemic inflammation.	**- -**	**- -**	Freitas et al., 2020 (58)	Kemmerling et al., 2019 (62)
Jaundice	Mild to moderate hyperbilirubinemia caused by liver dysfunction or inflammation.	Uncommon; reported in CZS.	Commonly observed in congenital CD.	Morilla Guzmán et al., 2017 (209)	Edwards et al., 2021 (210)
Anemia, thrombo-cytopenia	Due to systemic placental inflammation.	Cytopenias described in neonates with CZS.	**- -**	Vouga et al., 2018 (211)	Edwards et al., 2021 (210)
Brain damage	Neurological involvement can occur in both infections, although the characteristic manifestations differ.	Microcephaly, commonly observed in CZS. Collapsed skull, subcortical calcification.	Meningoencephalitis with seizures and neurological damage.	Freitas et al., 2020 (58)	Chancey et al., 2023 (21)
Liver damage	Hepatomegaly or liver inflammation in severe cases.	Uncommon; case reports in CZS.	Hepatosplenomegaly.	Freitas et al., 2020 (58)	Edwards et al., 2021 (210)
Cardiac alterations		Uncommon; transient dysfunction/cardiomegaly reported.	Cases of myocarditis and sometimes heart failure noted.	Angelidou et al., 2018 (64) Orofino et al., 2018 (212)	Chancey et al., 2023 (21)
Pulmonary alterations		Pulmonary hypoplasia reported in severe cases of CZS	Pneumonitis with respiratory distress in some cases.	Guerrero et al., 2023 (66)	Chancey et al., 2023 (21)
Generalized edema	May result from multi-organ damage.	Hydrops fetalis reported in severe congenital ZIKV cases	Hydrops fetalis reported sometimes.	Sarno et al., 2016 (67)	Gonzalez et al. (69)
Structural abnormalities		Subcortical calcification (neuroimaging, autopsy) reported.	Acute colonic dilation reported *	Alvarado et al., 2017 (63)	de Almeida et al., 1986 (213) Costa-Pinto et al., 2001 (214)
Ocular involvement	Ocular abnormalities are prominent in ZIKV; uncommon but reported in congenital CD.	Maculopathy and retinal scarring with pigmentary changes characteristic of CZS	Uncommon; limited/isolated reports	Alvarado et al., 2017 (63)	Berberian et al., 2013 (68)

CZS, Congenital Zika Syndrome; CD, Chagas disease.

indicate manifestations are described under the “*Common to both ZIKV and T. cruzi*” column.

* Acute colonic dilation due to direct parasitic invasion has been reported in congenital CD (213), and chronic digestive mega-syndrome develops in later stages of infection.

This table summarizes the principal neonatal symptoms observed in CZS and *T. cruzi* infections, highlighting overlapping and pathogen-specific features. “Common to both” refers to findings described in both congenital infections, usually linked to systemic inflammation or placental dysfunction. The “Specific to ZIKV” and “Specific to *T. cruzi*” columns indicate clinical manifestations uniquely or predominantly reported for specific pathogen, based on human neonatal and placental studies.

## 5 Immunological features of congenital infection and impact on neonatal immune development

Understanding the ontogeny of the fetal immune system provides essential context for interpreting how congenital infections (such as those caused by ZIKV and *T. cruzi*) may alter immune maturation and postnatal immunity. During pregnancy, the maternal-fetal interface establishes a finely tuned balance between immune tolerance and defense. This equilibrium is crucial for maintaining pregnancy while permitting limited immune responsiveness to microbial threats. Disturbance of these processes by congenitally transmitted pathogens can permanently reprogram immune development, affecting the neonate’s ability to respond to infections and vaccines. We summarize the normal trajectory of fetal immune development to provide a framework for interpreting infection-induced immune alterations.

During gestation in humans, the fetal immune system develops in successive waves. Initially, primitive hematopoiesis in the yolk sac generates erythroblasts and macrophages, with the latter migrating to developing organs, including the pancreas, by 6-weeks of gestation (88–91). The aorta–gonad–mesonephros region produces the first clusters of hematopoietic stem cells (HSCs), which later colonize the fetal liver, the primary hematopoietic organ during 5- to 20-weeks of gestation. At this stage, liver produces pre-B lymphocytes (~7-weeks) and pre-T lymphocytes (8-to-9-weeks). Mesenchymal cells in the fetal skin and kidneys share developmental similarities with those in the liver.

Fetal mast cells can bind IgE receptors and contribute to early immune reactivity (92). Fetal natural killer (NK) cells emerge early and exhibit functional immaturity, but their numbers are higher relative to adaptive lymphocytes (93, 94). Innate lymphoid tissue inducer (LTi) cells, critical for secondary lymphoid organogenesis, recruit additional lymphocytes through crosstalk with stromal cells (95–97). By ~8-weeks of gestation, the thymus, bone marrow, and spleen initiate T, B, and NK cell maturation, although pattern recognition receptor signaling (e.g., via TLRs or RIG-I/MAVS) remains attenuated until late gestation, preserving maternal–fetal tolerance (98). This baseline immune architecture provides the context for understanding how congenital pathogens such as ZIKV and *T. cruzi* exploit or disrupt fetal immune pathways.

### 5.1 Congenital ZIKV infection and immune modulation

The placenta not only regulates gas and nutrient exchange but also establishes a finely tuned immunological environment that enables fetal protection from infection without compromising the pregnancy. This balance relies on controlled activation of maternal innate and adaptive immune populations-including macrophages, dendritic cells, NK cells, and regulatory T cells-which maintain an anti-inflammatory, homeostatic milieu at the maternal–fetal interface (99–101). Still, ZIKV infection can disrupt this equilibrium by altering decidual immune-cell composition and trophoblast–immune interactions. Experimental studies indicate that decidual NK cells and CD8^+^ T cells contribute to viral control at the maternal–fetal interface. In a rhesus macaque model, ZIKV infection altered decidual immune-cell composition and reduced activated and cytotoxic memory T-cell populations, whereas trophoblast studies demonstrated that decidual NK cells can recognize and eliminate ZIKV-infected trophoblasts (102, 103). Together, these and other observations support that ZIKV disrupts placental immune homeostasis by reshaping decidual immune-cell composition and altering the interactions between trophoblasts and antiviral effector cells (27, 53, 104).

At the cellular and molecular levels, placental restriction of ZIKV is strongly influenced by the MAVS–type I interferon (IFN-I) axis (34). ZIKV has evolved mechanisms that interfere with antiviral signaling, including disruption of RIG-I/MAVS-dependent responses by viral non-structural proteins (105). Nevertheless, placental IFN-I signaling remains functionally important: experimental disruption of MAVS or IFNAR receptor increases placental susceptibility to ZIKV, whereas sustained IFN-I activation can also contribute to placental injury and adverse fetal outcomes (33). Thus, IFN-I responses at the maternal–fetal interface can exert both antiviral and immunopathological effects depending on their magnitude, timing, cellular source, and gestational context. Type III interferons constitute another important component of placental antiviral defense. Primary human trophoblasts can constitutively produce IFNλ-1/IL-29, conferring autocrine and paracrine resistance to ZIKV (31), whereas first-trimester placental tissues display delayed induction of IFN-β, IFNλ-2/3, and multiple interferon-stimulated genes following infection (32), indicating that ZIKV does not generate a uniformly suppressed interferon environment; rather, interferon responses vary according to placental cell type and gestational stage, and delayed or incomplete activation may provide a temporal window for viral replication and dissemination (31, 32).

ZIKV infection may also disrupt placental angiogenic balance. Studies have reported downregulation of VEGF and impaired vascular remodeling in infected villi, further compromising trophoblast integrity and local perfusion. In parallel, ZIKV activates the NLRP3 (NOD-like receptor family pyrin domain-containing 3) inflammasome in trophoblasts and Hofbauer cells, a mechanism increasingly recognized as central to placental pathology. NLRP3 activation results in apoptosis-associated speck-like protein containing a CARD (ASC) – caspase 1 complex assembly and IL-1β and IL-18 activation, triggering pyroptosis (36, 56, 59, 106). Severe ZIKV infection has been associated with marked inflammatory remodeling and endothelial injury at the maternal–fetal interface, contributing to placental dysfunction and impaired vascular integrity (**Figure 2**) (27, 107). This inflammatory axis may contribute to villitis, syncytial disruption, fibrinoid necrosis, and microvascular thrombosis, processes that compromise placental perfusion and contribute to intrauterine growth restriction.

**Figure 2:**
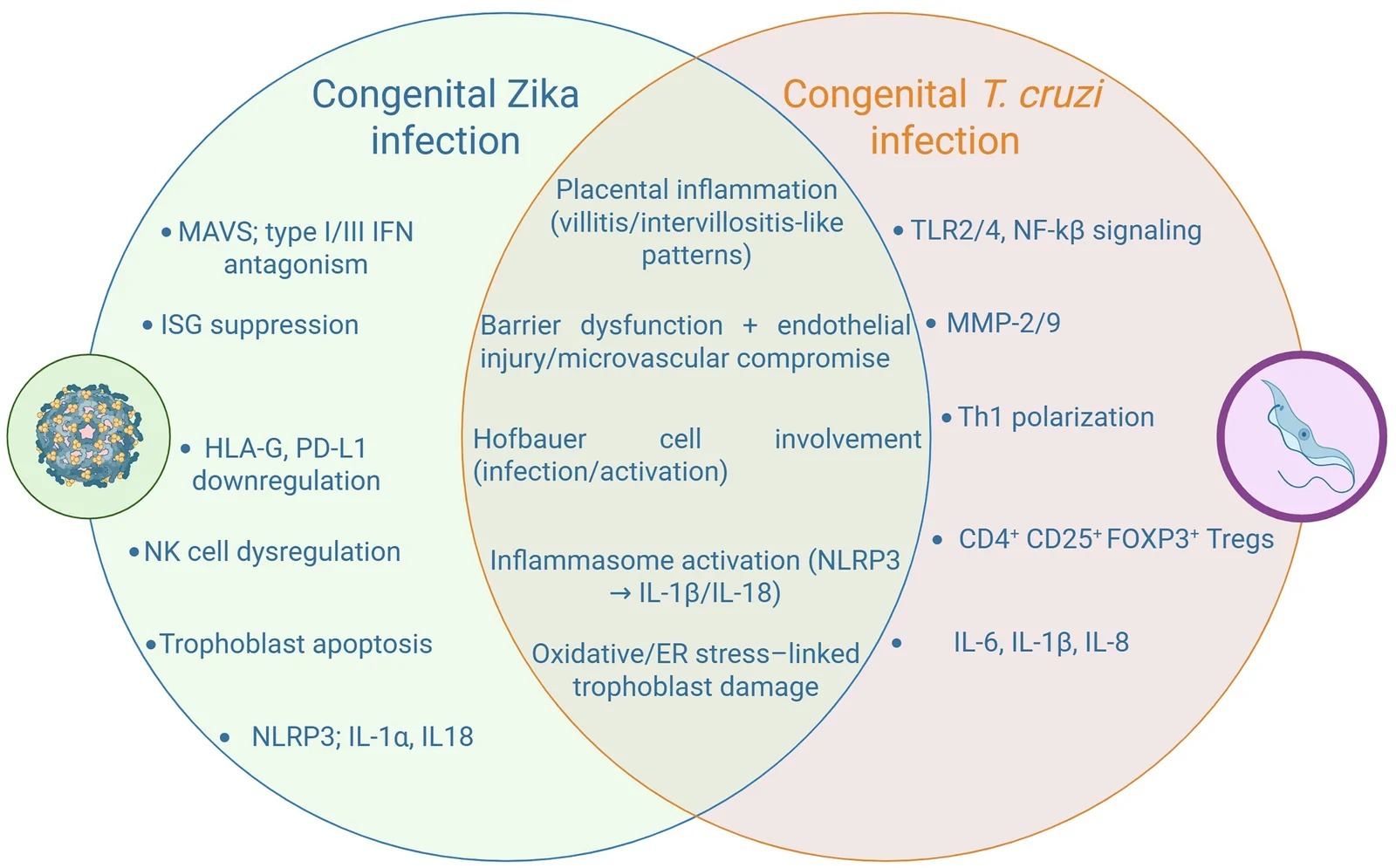
Integrated immunological landscape of congenital ZIKV and Trypanosoma cruzi infections. Conceptual summary of pathogen-specific and shared immune perturbations at the maternal-fetal interface, highlighting distinct antiviral and antiparasitic immune features alongside convergent inflammatory, vascular, and regulatory pathways implicated in placental dysfunction and fetal immune programming during congenital infection. Created in BioRender. Rodriguez salazar, C. A. (2026) https://BioRender.com/0jl99gs.

Growing evidence indicates that ZIKV perturbs molecular pathways involved in maternal–fetal immune tolerance. In trophoblast models and primary extravillous trophoblasts, ZIKV-induced cellular stress has been associated with reduced HLA-G expression, potentially increasing susceptibility to decidual NK-cell-mediated cytotoxicity. In contrast, recent evidence indicates that ZIKV can upregulate PD-L1 expression, including in trophoblast-like cells, suggesting that different immune-regulatory checkpoints may be modulated in distinct directions during infection. Experimental PD-L1 blockade enhances antiviral T-cell responses and reduces viral burden, supporting a potential role for this pathway in limiting antiviral immunity. Notably, genetic analyses in mother–infant pairs have identified HLA-G 3′UTR variants associated with susceptibility to congenital ZIKV transmission, further supporting the relevance of HLA-G-dependent tolerance mechanisms at the maternal–fetal interface. Collectively, these findings indicate context-dependent remodeling, rather than uniform suppression, of immune-regulatory pathways during ZIKV infection (55, 102, 108, 109) (**Figure 2**).

Beyond these placental immune alterations, the fetal immune compartment shows evidence of early activation upon ZIKV exposure. Detection of ZIKV-specific IgM or IgA in cord blood and amniotic fluid reflects antigen exposure *in utero*. Newborns with congenital infection exhibited a Th1-skewed cytokine profile driven by IFN-γ and TNF-α with diminished IL-10, suggesting chronic immune activation in the absence of balanced regulatory maturation (8). This profile is consistent with studies reporting elevated inflammatory mediators in cord blood and early-life peripheral monocytes following prenatal ZIKV exposure (110).

### 5.2 Congenital *T. cruzi* infection and neonatal immunity

Congenital *T. cruzi* infection arises from the interplay between high maternal parasitemia, pregnancy-associated immune modulation, and parasite-driven disruption of the placental barrier. Studies in human placental explants showed that *T. cruzi* infects STB, CTB, and villous stroma, activating TLR2/TLR4-NF-κB pathways and inducing proinflammatory cytokines/chemokines (e.g., IL-1β, TNF-α, IL-6, IL-8) that recruit maternal and fetal inflammatory cells. Beyond TLR-mediated activation, several studies have shown that *T. cruzi* also activates the NLRP3 inflammasome in macrophages, promoting caspase 1 mediated IL-1β maturation during acute infection. Although direct evidence in human placental tissue is limited, NLRP3 activation may contribute to local inflammatory amplification during congenital transmission (56, 106). It can, thus, be surmised that the inflammatory cascades, along with focal STB lesions, may permit parasite traversal into the fetal compartment (**Figure 2**).

Additionally, *T. cruzi* infection increases the expression of matrix metalloproteinases such as MMP-2 and MMP-9 and promotes endothelial activation, contributing to extracellular matrix degradation and altered placental angiogenesis (111). In parallel, placental trophoblasts mount an early but incomplete regulatory response, producing IL-10 and TGF-β to restrict excessive inflammation and preserve tissue integrity; however, the magnitude of proinflammatory signaling typically overwhelms these anti-inflammatory pathways, particularly in mothers with high parasitemia (19, 54).

Once the parasite reaches the fetus, neonatal immunity is prematurely activated. Congenitally infected newborns exhibit markedly elevated IFN-γ, TNF-α, IL-1β, IL-6, and IL-8 levels, reflecting a strong Th1-skewed profile (22, 57). Still, fetal CD8^+^ T cells and NK cells are identifiable in cord blood but are functionally delayed, displaying limited cytotoxic granule release and reduced capacity to clear intracellular amastigotes (112). This ineffective immune response contributes to parasite persistence and bystander damage to fetal tissues, thereby increasing susceptibility to postnatal myocarditis (**Figure 2**).

Crucially, the anti-inflammatory axis constituted by IL-10, TGF-β, and regulatory T cells (Tregs) is dysregulated in congenital infection. Infected neonates show reduced IL-10 secretion and decreased frequencies of CD4^+^CD25^+^FoxP3^+^ T regulatory cells, resulting in net increase in systemic inflammation (87). Even when IL-10 and TGF-β are produced by fetal monocytes or trophoblasts, their regulatory effect is insufficient to restrain the robust Th1 and innate cytokine milieu. This imbalance mirrors the findings at the placental interface, where anti-inflammatory mediators are present but are overridden by parasite-driven proinflammatory and tissue-disruptive pathways (**Figure 2**).

Interestingly, uninfected newborns born to seropositive mothers also show heightened baseline inflammatory cytokines and stronger responses to TLR stimulation, indicating *in utero* antigenic sensitization without productive infection (113). These heightened responses suggest early innate immune imprinting driven by exposure to maternal parasite antigens, potentially altering monocyte and dendritic cells programming in early life. In these neonates, the anti-inflammatory pathways remain partially intact, suggesting a compensatory regulatory tone that limits immunopathology compared with infected newborns.

Parasite-derived extracellular vesicles (EVs) may further intensify the immune imbalance during pregnancy. EVs released by *T. cruzi* are shown to activate TLR2/TLR4, induce ROS, promote apoptosis, and disrupt tight-junction integrity in trophoblasts, decidual macrophages and other cells (114, 115). Acute *T. cruzi* infection also engages complement pathway, with activation of C3 and C5 convertases contributing to inflammation and parasite control (116). Although trophoblasts attempt to counteract this inflammatory damage via IL-10/TGF-β secretion, the combined effect of EV-driven inflammation and mechanical disruption can overwhelm the regulatory programs. Though further studies in human placental tissue are needed, it is likely that congenital exposure to *T. cruzi* is presented with an imbalanced immune response that fails to prevent parasite invasion and immune injury to fetus.

In summary, congenital *T. cruzi* infection exposes the fetus to a dysregulated immunological environment in which pro- and anti-inflammatory pathways coexist but fail to restore homeostasis. Congenital ZIKV infection likewise produces a heterogeneous immune landscape, with antiviral interferon responses, immune-checkpoint remodeling, and inflammatory injury varying according to cell type and gestational stage. Ultimately, both pathogens reshape placental and neonatal immunity through distinct but partially overlapping mechanisms that may contribute to pathogen persistence and fetal tissue injury.

### 5.3 Hypothesis-generating framework for immunological interplay during co-infection

Because no studies have directly evaluated ZIKV–*T. cruzi* co-infection at the maternal–fetal interface, the framework below extrapolates from pathogen-specific mechanisms and is intended to generate testable hypotheses rather than describe established consequences of dual infection.

Interferon signaling provides a particularly relevant mechanistic bridge between the two infections. ZIKV can induce IFN-I/III responses in placental cells while simultaneously antagonizing interferon induction and downstream signaling through viral non-structural proteins, potentially generating spatially heterogeneous antiviral states at the maternal–fetal interface (31, 32, 53, 104, 105, 117). Such heterogeneity provides a rationale for testing whether ZIKV-conditioned placental environments differentially restrict or facilitate *T. cruzi* infection.

Hofbauer cells provide a particularly relevant cellular context for testing this hypothesis because they are permissive to ZIKV infection and occupy the villous stromal compartment also involved in placental *T. cruzi* infection (27, 53, 104). One testable hypothesis is that ZIKV-induced IFN-I signaling in neighboring placental macrophages could enhance antiparasitic activity and restrict *T. cruzi* replication, consistent with experimental evidence outside the placenta showing that IFN-I can promote nitric oxide production and contribute to parasite control (118, 119). Conversely, ZIKV-mediated attenuation of IFN-I signaling within infected cells could create locally permissive niches in which antiparasitic responses are weakened, potentially favoring *T. cruzi* persistence. A further alternative is that sustained or dysregulated IFN-I signaling could amplify placental inflammation and tissue injury without necessarily improving parasite control, as context-dependent detrimental effects of IFN-I have also been described during *T. cruzi* infection (118–120).

A second hypothesis is that simultaneous infection could generate a placental microenvironment more permissive to transplacental dissemination through convergent inflammatory, vascular, and tissue-remodeling mechanisms. ZIKV-associated trophoblast and endothelial injury, together with altered VEGF-associated signaling, could weaken structural and vascular barriers, whereas *T. cruzi*-induced inflammasome activation, cytokine production, complement signaling, and MMP-mediated extracellular-matrix remodeling could further compromise villous architecture and endothelial integrity (29, 36, 56, 59, 106, 107, 116). Under this scenario, combined infection could increase endothelial permeability, villous hypoxia, or access to fetal vascular compartments beyond that produced by either pathogen alone. Conversely, an intensified inflammatory response might restrict replication of one pathogen while simultaneously exacerbating placental tissue injury. These alternative outcomes provide experimentally distinguishable models of synergistic versus antagonistic interaction.

Beyond the magnitude of acute barrier injury, a third hypothesis is that dual infection could compromise trophoblast repair and placental resilience after infection-associated damage. ZIKV infection alters trophoblast migration, metabolism, and cellular homeostasis, whereas *T. cruzi* disrupts villous architecture, trophoblast turnover, and differentiation-associated pathways (29, 47–49, 54). Convergence of these effects could impair restoration of the trophoblast barrier, allowing structural defects to persist even without increased replication or transplacental transmission of either pathogen. Such defective placental repair could contribute to sustained placental insufficiency, impaired maternal–fetal exchange, and adverse fetal growth independently of pathogen burden.

A fourth hypothesis involves disruption of maternal–fetal immune tolerance. ZIKV-associated changes in HLA-G expression and PD-1/PD-L1 signaling (55, 108, 109), together with alterations in IL-10/Treg-mediated regulation during *T. cruzi* infection (87), could generate spatially heterogeneous immune states within the placenta. Reduced trophoblast HLA-G could enhance NK-cell-mediated cytotoxicity and tissue injury, whereas increased PD-L1 signaling or compensatory regulatory pathways could suppress antiviral or antiparasitic effector responses in other placental compartments. Dual infection could therefore produce a mosaic of excessive inflammation and local immune suppression, potentially favoring both placental damage and pathogen persistence.

A fifth hypothesis concerns fetal immune programming. One possibility is that combined inflammatory signaling enhances prenatal priming of fetal monocytes and dendritic cells, resulting in heightened innate responsiveness after birth. Alternatively, prolonged antigenic and cytokine exposure could promote regulatory adaptation or immune hyporesponsiveness. Experimental and human studies showing persistent effects of prenatal inflammation on fetal hematopoietic and innate immune development provide biological support for testing these possibilities (121–123).

It is important to note that the infection sequence, gestational stage, cellular compartment, and the magnitude and duration of local interferon signaling may modulate each of these proposed interactions. In the epidemiologically plausible setting of chronic maternal *T. cruzi* infection followed by acute ZIKV infection, pre-existing parasite-driven inflammatory and regulatory responses could shape the subsequent placental response to ZIKV. Conversely, ZIKV infection preceding *T. cruzi* exposure could transiently modify interferon signaling, trophoblast integrity, and macrophage activation before parasite arrival. Sequential and simultaneous infections may therefore generate biologically distinct placental environments, making infection order and timing important cross-cutting determinants of the proposed interactions.

The hypotheses proposed above could be tested using primary Hofbauer cells, human placental explants, and placental organoid systems. Studies in experimental models varying the order and interval of pathogen exposure would be particularly informative for distinguishing whether ZIKV-induced antiviral priming restricts subsequent *T. cruzi* infection or whether prior parasite-driven inflammation increases susceptibility to ZIKV-mediated placental injury. Integrated measurements of pathogen burden, IFN-I/III signaling, inflammasome activation, trophoblast migration and barrier-repair capacity, endothelial permeability, HLA-G/PD-L1 expression, cytokine profiles, and fetal immune-cell phenotypes would allow additive, synergistic, and antagonistic interaction models to be experimentally distinguished.

## 6 Challenges and opportunities for the development of vaccines against ZIKV and *T. cruzi*

The development of vaccines against ZIKV and *T. cruzi* faces substantial challenges, including the biological and immunological complexity of each pathogen and limited prioritization in global health agendas. Compared to other diseases like malaria, tuberculosis, or HIV, both pathogens receive insufficient funding and attention (10, 124–126). Although ZIKV has caused outbreaks with severe neurological and congenital consequences, its relatively low and sporadic prevalence has hindered sustained investment in vaccine development. Similarly, *T. cruzi* primarily affects human populations in Latin America, and its chronic disease course contributes to low visibility in international health policies. These factors collectively delay progress in therapeutic and preventive strategies. Overcoming these barriers will require sustained long-term investment, strengthened international collaboration, and aggressive basic and translational research to better characterize the biology, immunogenicity, and correlates of protection for both pathogens (10, 125, 127).

One of the major challenges in ZIKV vaccine development lies in antibody-dependent enhancement (ADE) phenomenon, in which pre-existing, non-neutralizing antibodies from prior flavivirus infections (e.g., Dengue) facilitate ZIKV entry into host cells, increasing viral replication and disease severity. This risk complicates vaccine design, requiring approaches that elicit strong protective immunity without triggering ADE (10, 128). Additionally, minor genetic variations in envelope and NS1 viral proteins among ZIKV strains from Africa, Asia, and Americas, can affect cross-protective immunity and vaccine efficacy. The sporadic and low incidence of ZIKV outbreaks, along with limited diagnostic capacity for febrile syndromes in endemic areas, further hinders vaccine development by complicating the execution of large-scale clinical trials (129).

*T. cruzi* has multiple developmental stages, and it colonizes diverse host tissues to evade the immune system, particularly during indeterminate-to-chronic infection, which leads to low-grade but persistent inflammation and tissue damage, especially in the heart and gastrointestinal tract. Vaccine development against *T. cruzi* is hindered by its complex life cycle, as an effective vaccine must target multiple parasite stages. Furthermore, high genetic diversity of *T. cruzi*, reflected in its discrete typing units (DTUs), poses an additional challenge, as strain-specific immunity may limit vaccine coverage (126). Although promising vaccine candidates and therapies are emerging, a major obstacle remains: the absence of preclinical models that fully recapitulate human CD, especially chronic immunopathology. This gap undermines the ability to predict vaccine efficacy in humans accurately and slows the progress toward a broadly protective and effective vaccine (82, 83, 126).

### 6.1 Candidate vaccines for ZIKV

Multiple ZIKV vaccine platforms have reached clinical evaluation, although their stages of development differ substantially and no vaccine has yet been licensed (**Table 2**). Inactivated-virus candidates, including ZPIV, PIZV/TAK-426, and VLA1601, have demonstrated acceptable safety and immunogenicity in early-phase human studies. ZPIV and VLA1601 completed Phase 1 evaluation, whereas a planned Phase 2 study of PIZV/TAK-426 was withdrawn before participant enrollment, illustrating how the decline and unpredictability of ZIKV circulation has complicated further clinical development (128–133).

**Table 2:** Overview of candidate vaccines against the Zika virus (ZIKV). Summary of vaccine platforms, clinical development stages, preclinical models, and reported fetal protection outcomes.

Candidate vaccine	Platform	Clinical development status	Preclinical model	Fetal protection	Key findings	Key references
ZPIV	InV	Phase 1	hSTAT2KI mice, NHPs	Yes	Prevented placental/fetal infection; strong neutralizing antibodies	Kim et al., 2024 (130)
PIZV/ TAK-426	InV	Phase 1*	NHPs	Not reported	Induced neutralizing antibodies; protected NHPs from infection	Han *et a.l* 2021 (215)
VLA1601	InV	Phase 1	Rodents	Not reported	Safe and immunogenic	Wressnigg et al., 2024 (133)
rZIKV/D4Δ30-713	Live attenuated (DENV-4 backbone)	Phase 1	Murine	Not assessed	Acceptable safety and tolerability in Phase 1; modest seroconversion; candidate considered over-attenuated	Zhong et al., 2022 (136)
E80/E90	Subunit (ZIKV E protein)	Preclinical	Mice, NHPs*	Not reported	Induced strong humoral and cellular responses	Zhu *et a.l* 2018 (216).
VRC5283 /VRC5288	DNA (prM-E)	Phase 2/2B/ Phase 1	Mice, NHPs*	Yes	Reduced placental damage and fetal infection	Gaudinski *et a.l* 2018 (134, 166)
GLS-5700	DNA (prM-E)	Phase 1	Mice	Not reported	Safe; induced neutralizing antibodies	Tebas *et a.l* 2021 (135)
mRNA-1325 /mRNA-1893	mRNA	Phase 1 / Phase 2	Rodents	Not reported	Safe; elicited robust antibody responses regardless of prior flavivirus exposure.	Bollman *et a.l* 2023 Essink *et a.l* 2023, (217, 218)
ChAdOx1 ZIKV	Ad	Phase 1	Mice	Not reported	Strong immunity; no ADE in presence of DENV antibodies	Lopez C. *et a.l* 2018 (219)
MV-ZIKA-RSP	MV	Phase 1	Mice	Not reported	Safe; induced neutralizing antibodies	Themis Bioscience Gmbh (220)
Ad26.ZIKV.001	Ad26	Phase 1	Rodents	Not reported	Immunogenic and safe	Salisch *et a.l* 2021 (221)
MVA-ZIKV-NS1	MVA	Preclinical	Rodents	Not assessed	Protective antibodies without inducing ADE	Brault *et a.l* 2017 (222)

InV, Inactivated virus; MVA, Modified Vaccinia Ankara vector; Ad, Adenoviral vector; MV, Measles virus vector; NHPs, non-human primates; ADE, antibody-dependent enhancement; prM, premembrane; E, Envelope protein.

*The planned Phase 2 PIZV/TAK-426 study (NCT05469802) was withdrawn before enrollment.

Data are based on published preclinical and clinical studies and trial-registry records available through September 2026.

Among nucleic acid vaccines, VRC5283 represents the most extensively clinically evaluated candidate. Following Phase 1 testing, it advanced to and completed a large Phase 2/2B randomized trial, whereas the related VRC5288 candidate remained at Phase 1 evaluation. Similarly, mRNA-1325 and mRNA-1893 should be distinguished by their respective development stages: mRNA-1325 completed Phase 1 evaluation, while mRNA-1893 subsequently completed a randomized Phase 2 study involving 808 adults in flavivirus-endemic and non-endemic settings. Both mRNA platforms elicited neutralizing antibody responses in Phase 1 studies, while the Phase 2 mRNA-1893 study further evaluated dose, safety, and immunogenicity (128, 134–136). Other nucleic acid vaccines such as GLS-5700, MV-ZIKA-RSP, Ad26.ZIKV.001, ChAdOx1 ZIKV, and rZIKV/D4Δ30–713 have undergone Phase 1 evaluation. Nevertheless, progression to a clinical phase should not be interpreted as evidence of established protection against congenital ZIKV infection. Most human trials have focused on safety and immunogenicity in non-pregnant adults, while evidence for prevention of placental or fetal infection derives largely from animal models. The episodic epidemiology of ZIKV remains a major obstacle to conducting conventional efficacy trials and to defining correlates of protection relevant to pregnancy.

### 6.2 Candidate vaccines for *T. cruzi*

Vaccine development against *T. cruzi* remains at the preclinical stage and encompasses multiple antigenic targets, delivery platforms, and intended clinical applications (**Table 3**). Importantly, candidate vaccines can be broadly distinguished as prophylactic strategies, designed to prevent infection or improve early parasite control, and therapeutic strategies, intended to enhance immune control after infection has already been established. This distinction is particularly relevant to congenital Chagas disease because vaccination before pregnancy could theoretically reduce maternal parasite burden and thereby decrease transmission risk, whereas therapeutic vaccination of chronically infected individuals addresses a different clinical objective.

**Table 3:** Overview of candidate vaccines against *T. cruzi*.

Candidate vaccine	Platform	Intended use	Development stage	Preclinical model	Pregnancy/congenital-transmission evidence	Key findings	Key references
Tc24 (rTc24)	Recombinant protein	Prophylactic	Preclinical	Mice	Not assessed	Partial protection	Martinez C. et al., 2015 (137)
TSA-1	DNA vaccine	Prophylactic	Preclinical	Mice	Not assessed	Partial protection	Wizel et al.*, 19*98 (151)
ASP-2	Recombinant protein	Prophylactic	Preclinical	Mice	Not assessed	CD8^+^ T-cell-dependent protection and reduced acute parasite burden	Barbosa et al., 2013 (139)
Cruzipain	Recombinant protein	Prophylactic	Preclinical	Mice	Not assessed	Protective Th1-skewed response with reduced parasitemia	Schnapp et al.*, 20*02 (152)
ASP-2 and TS (TRASP)	Chimeric protein	Prophylactic	Preclinical	Mice	Not assessed	TRASP protein induced robust immunity, zero lethality, and low parasite burden.	Castro et al., 2023 (138)
TSA-1/Tc24	DNA-based	Prophylactic and therapeutic	Preclinical	Dogs	Not assessed	Induced humoral responses, Reduces parasitemia and clinical severity	Quijano H. et al., 2013 (153)
TcSP/TcSSP4 (DNA)	DNA-based	Prophylactic	Preclinical	Dogs	Not assessed	Induces IgG/Th1 immunity and moderate protection.	Arce F. et al., 2013 (154)
TcTASV	Recombinant protein baculovirus-based delivery	Prophylactic	Preclinical	Mice	Not assessed	Humoral and cellular responses	Masip et al., 2024 (150)
TcG1/TcG2/TcG4 (DNA/Protein/MVA)	Prime/Boost (DNA/Protein/MVA)	Prophylactic	Preclinical	Mice	Not assessed	Reduced acute parasite burden and chronic cardiac pathology; induction of protective Th1/CD8^+^ T-cell responses in experimental models.	Gupta et al., 2012 (155)
TcG2/TcG4 nano2/4 — pregnancy model	Nanoplasmid DNA vaccine	Prophylactic	Preclinical	Mice	Pregnancy outcomes assessed; reduction in congenital transmission not established	Improved placental integrity, reduced maternal/placental inflammatory pathology, and improved fetal survival and pregnancy outcomes.	Rios et al., 2023 (170)
TcG2/TcG4	Nanoplasmid DNA vaccine	Therapeutic	Preclinical	Mice	Not assessed	Reduced parasite persistence and cardiac inflammatory/fibrotic pathology	Lokugamage et al., 2020 (223)

Summary of vaccine platforms intended use, development stage, preclinical models, and available evidence relevant to pregnancy and congenital transmission. Prophylactic or therapeutic classification refers to the intended use of the specific vaccine formulation evaluated rather than to the antigen itself, because some antigens have been investigated in both settings.

Among prophylactic approaches, recombinant protein and nucleic acid-based vaccines targeting antigens such as Tc24, TSA-1, ASP-2, cruzipain, TcSSP4, TcSP, and TcTASV have elicited parasite-specific humoral and cellular responses and varying degrees of protection in experimental models (137–154). Recombinant and chimeric antigen formulations, including ASP-2/trans-sialidase combinations, have demonstrated immunogenicity and partial protection, whereas DNA- and viral vector-based approaches have generally aimed to enhance Th1 and cytotoxic T-cell responses required for control of intracellular parasite stages. These studies collectively demonstrate the feasibility of vaccine-induced control of *T. cruzi* while also highlighting variability in protective efficacy according to antigen composition, platform, parasite strain, and experimental model.

Multi-antigen strategies based on TcG antigens provide another example of this approach. TcG1/TcG2/TcG4 and subsequently simplified TcG2/TcG4 formulations, delivered using DNA, recombinant protein, modified vaccinia Ankara vectors, or nanoplasmid-based platforms, have reduced acute parasite burden and chronic cardiac pathology in murine models, with protective responses associated predominantly with Th1 and CD8^+^ T-cell immunity (155–159). Related studies in canine models have provided additional preclinical evidence of immunogenicity and parasite control. Importantly for the context of congenital infection, a TcG2/TcG4 nanovaccine has also been evaluated in pregnant mice; this pregnancy-specific evidence is discussed separately in Section 6.3.

Therapeutic vaccination represents a distinct strategy for individuals with established *T. cruzi* infection. Experimental Tc24/TSA-1 and TcG2/TcG4 therapeutic vaccines have been evaluated alone or in combination with benznidazole, with studies reporting reductions in parasite burden and cardiac pathology in animal models (145–148, 160–165). Vaccine-linked chemotherapy is of particular interest because it seeks to combine parasite-directed pharmacological activity with vaccine-induced cellular immunity; however, these approaches remain experimental, and their efficacy in offering clinically meaningful outcomes in humans has not been established.

Overall, the *T. cruzi* vaccine field comprises multiple but still preclinical strategies, and no candidate has yet established clinical efficacy in humans. Direct comparison among platforms is complicated by differences in parasite strains, challenge protocols, animal models, immunization regimens, and outcome measures. Consequently, current evidence does not support prioritization of a single antigen or platform for prevention of congenital transmission. Progress toward clinical translation will require standardized preclinical endpoints, broader evaluation across parasite genetic diversity, characterization of correlates of protection in humans, and, for congenital disease prevention, dedicated studies addressing vaccination before or during pregnancy and its effects on maternal parasitemia, placental infection, fetal safety, and congenital transmission.

### 6.3 Special considerations for vaccines against congenital infections

Maternal immunization could potentially protect the maternal–fetal unit through improved maternal pathogen control and transplacental transfer of vaccine-induced IgG antibodies; after birth, breast milk may provide additional mucosal protection through secretory IgA and other immune factors. For *T. cruzi*, however, the extent to which maternal antibodies from vaccination would prevent congenital transmission remains uncertain, given the parasite’s intracellular lifecycle and its capacity for immune evasion. Early postnatal interventions are essential to mitigate long-term immune dysfunctions associated with congenital infections. The development of vaccines against these pathogens must prioritize safety during pregnancy. Live-attenuated vaccines and viral vector-based platforms require rigorous evaluation to ensure they do not adversely affect fetal development. The immunogenicity and safety profiles of these vaccines must be carefully optimized to balance protective maternal immunity while minimizing potential risks to the fetus (128).

Recent studies have demonstrated the potential of various vaccine platforms, including DNA-based (166), inactivated virus (167), live-attenuated (168), and mRNA vaccines (169), in preventing congenital ZIKV infection. Animal models have shown promising results, evidenced by reduced maternal viremia, lower fetal infection rates, and fewer congenital abnormalities. However, translating these findings into human clinical trials continues to be a challenge due to the episodic and unpredictable nature of ZIKV outbreaks and ethical considerations involving pregnant populations.

Pregnancy-specific evidence for *T. cruzi* vaccination remains particularly limited. To our knowledge, only one published study has directly evaluated a candidate vaccine in pregnant mice. In that study, a TcG2/TcG4 nanovaccine was well tolerated and modulated maternal and placental T-cell responses, reduced *T. cruzi*-associated inflammatory tissue damage, improved placental integrity and efficiency, and increased fetal survival and favorable pregnancy outcomes. However, a reduction in congenital transmission was not established because fetal infection was infrequent irrespective of vaccination status (170). Although these findings provide proof-of-concept that vaccination can influence maternal–fetal outcomes in experimental *T. cruzi* infection, confirmation in additional pregnancy models, parasite strains, and vaccine platforms is required before conclusions regarding translational potential can be drawn.

Finally, ongoing research remains essential to the development of safe and effective vaccines against congenital ZIKV and *T. cruzi* infections. Progress toward clinical application will depend on coordinated efforts that integrate maternal–fetal immunology, standardized pregnancy-safe vaccine testing pipelines, and strengthened regulatory frameworks for trials involving pregnant populations.

## 7 Therapeutic approaches for ZIKV and *T. cruzi* infections

### 7.1 Therapeutic approaches for ZIKV infection

Currently, acute febrile syndrome caused by ZIKV is managed by supportive care, as no approved antiviral treatments are available. Supportive management includes rest, adequate hydration, and acetaminophen (paracetamol) for fever or pain. Aspirin and other non-steroidal anti-inflammatory drugs should generally be avoided until dengue has been excluded because of the potential risk of hemorrhage; medication use during pregnancy should additionally follow obstetric safety recommendations. Early diagnosis and close monitoring are essential, especially in pregnant individuals, due to the risk of neurological complications and congenital transmission (171). In severe cases such as ZIKV-associated Guillain-Barré Syndrome (GBS), treatment includes intravenous immunoglobulin (IVIg), and plasmapheresis to reduce inflammation and aid neurological recovery. Supportive management of cardiovascular and autonomic instability is often necessary, given the hemodynamic fluctuations associated with GBS. Neuropathic pain is managed with agents like gabapentin, pregabalin, or amitriptyline. As patients improve, rehabilitation therapy to restore mobility and muscle strength is offered. Long-term follow-up is needed due to persistent sequelae, including weakness, fatigue, and autonomic dysfunction (171, 172).

In pregnant women, management of ZIKV infection involves fetal monitoring and genetic counseling, particularly when complications like microcephaly are detected. If GBS occurs during pregnancy, IVIg is preferred over plasmapheresis, which carries additional risks, including hypotension, infections, hemorrhagic complications, and potential impacts on uterine perfusion that may compromise fetal health. Preventive strategies are also essential in endemic areas, where mosquito control measures (e.g., insect repellents, bed-nets) are recommended to reduce transmission risk during pregnancy (172).

In contrast to the vaccine pipeline, ZIKV antivirals have not advanced to clinical phase. For example, preclinical studies have identified Isobavachalcone and corosolic acid to interfere with viral replication through effects involving NS1/NS2A-associated metabolic pathways, and 6-bromo-1,2-naphthalenedione to inhibit the NS2B–NS3 protease complex. Another compound, SBI-0090799 disrupts formation of membranous replication compartments, and afatinib derivative L3 targets host HER2-associated signaling. Although several of these compounds have shown activity in both cell-based and animal models, their pharmacokinetics, pregnancy safety, and therapeutic efficacy in humans remain unknown (173–176). Drug-repurposing approaches have identified atovaquone, dabrafenib, and regorafenib with antiviral activity in experimental ZIKV systems (173, 177–192). The broad-spectrum antiviral nucleoside analog galidesivir has undergone Phase 1 safety and pharmacokinetic evaluation in healthy adults; however, this clinical study was not designed to evaluate anti–ZIKV efficacy, and no clinical efficacy against ZIKV has been established (193).

Overall, most small molecules discussed above have been evaluated only *in vitro* or in animal models for their anti-ZIKV efficacy. Prior regulatory approval of a repurposed drug for another indication should likewise not be interpreted as evidence of clinical efficacy against ZIKV. Thus, these agents currently represent mechanistic or translational leads rather than ZIKV-specific therapies for human use.

### 7.2 Therapeutic approaches for control of *T. cruzi* infection and Chagas disease

Treatment of *T. cruzi* infection primarily relies on benznidazole and nifurtimox. Conventional regimens generally use benznidazole at approximately 5–8 mg/kg/day, administered orally in divided doses for about 60 days, whereas nifurtimox is commonly administered at approximately 8–10 mg/kg/day in adolescents and adults, with higher weight-based doses used in younger children, generally for 60 days. Antiparasitic treatment is indicated for acute, congenital, and reactivated infections and is also recommended or considered in chronic infection based on age, disease stage, cardiac involvement, and individual risk–benefit assessment. Treatment is most effective when initiated early in infection, whereas efficacy decreases and adverse effects become more frequent with increasing duration of infection and age. In patients with established chronic infection, anti-parasite treatment has not been shown to reverse the established structural cardiac damage, and management of cardiac, gastrointestinal, or neurological complications remains an essential component of patient care (194–197).

Beyond the anti-parasite therapy, management of chronic Chagas cardiomyopathy includes treatment of cardiac complications and adjunctive cardioprotective strategies. Amiodarone may be used for the management of ventricular arrhythmias in selected patients, whereas statins such as atorvastatin have been investigated as adjunctive anti-inflammatory and cardioprotective therapy rather than as established anti–trypanosomal treatment (198). Separately, alternative benznidazole dosing strategies, including lower-dose and shortened regimens, have been evaluated with the aim of improving tolerability while maintaining parasitological response; however, their impact on long-term clinical outcomes and their role in routine practice remain under investigation (199).

New therapeutic strategies against *T. cruzi* include drug combinations, immunomodulators, nanomedicine, and gene therapies aimed at enhancing the efficacy and reducing toxicity of benznidazole and nifurtimox (200). New drugs have also been developed over the last two decades. For example, cyanotriazoles selectively inhibit kinetoplastid topoisomerase II, showing promise against *T. cruzi* and other kinetoplastid infections (201). Similarly, 3-cyanopyridine derivatives and pyrazole-thiadiazole-based compounds have demonstrated strong activity against intracellular amastigotes, although some exhibit trypanostatic activity (202, 203). Phenoxyhydrazine-thiazole derivatives showed dual parasiticidal and immunomodulatory effects in murine models, enhancing nitric oxide and proinflammatory cytokines (204). Natural products like coptisine hydrochloride, sesquiterpenoid-triazole hybrids, and nanocarrier systems (e.g., curcumin or silver nanoparticles) have shown potent antiparasitic effects with low toxicity (205–208). Taken together, these advances highlight the promise of integrated synthetic, natural, and nanotechnological approaches to improve treatment outcomes, particularly in the chronic stage of Chagas disease; however, further validation in standardized preclinical models and early-phase clinical studies is essential before any of these candidates can realistically replace current therapies.

## 8 Conclusions and perspectives

Congenital infections caused by ZIKV and *T. cruzi* remain significant public health challenges in Latin America, particularly because their capacity to breach the maternal–fetal interface results in lifelong clinical consequences. Although their biological strategies differ profoundly (viral neurotropism versus intracellular parasitism), both pathogens converge on key placental pathways involving trophoblast injury, dysregulated interferon signaling, and disruption of immune tolerance mechanisms. These shared points of vulnerability help explain their ability to establish congenital infection and highlight the placenta as a central determinant of fetal outcome. Still, synergistic, antagonistic, additive, or biologically negligible effects of these interactions remain to be determined experimentally.

In the therapeutic and preventive landscape, significant advances in vaccine development for both pathogens support continued investigation of maternal and preconception vaccination strategies, although concerns such as antibody-dependent enhancement for ZIKV and antigenic diversity for *T. cruzi* remain major obstacles. Parallel progress in antiviral discovery and anti-parasitic drug innovation offers important avenues for reducing vertical transmission and mitigating fetal damage, especially when integrated with early screening programs for pregnant individuals.

Looking forward, multidisciplinary efforts that integrate placental biology, immunology, epidemiology, and translational vaccine and drug development will be essential to close persistent gaps. Priority areas include establishing reliable diagnostic algorithms for dual exposure, identifying immune signatures predictive of congenital transmission, evaluating whether treatment of one pathogen modifies susceptibility to the other, and designing pregnancy-safe vaccines with demonstrated effects on fetal protection.

Ultimately, addressing congenital ZIKV and *T. cruzi* infections (and their potential overlap) requires coordinated action across clinical, public health, and research sectors. Such integration is essential to improve prevention, diagnosis, and management in populations disproportionately affected by these neglected yet impactful pathogens.
